# 1023. Case Study. Enhancing Therapy Discharge Communication Across Receiving Facilities

**DOI:** 10.1093/jbcr/irag033.472

**Published:** 2026-04-06

**Authors:** Ryan Willging, Abigale Heitmann, Sara Harwood, Xia Olig, Courtney Waldhuetter, Jennifer Arndt, Nicholas A Meyer

**Affiliations:** Ascension Columbia St. Mary's Milwaukee, Milwaukee, WI; Ascension Columbia St. Mary's Milwaukee, Milwaukee, WI; Ascension Columbia St. Mary's Milwaukee, Milwaukee, WI; Ascension Columbia St. Mary's Milwaukee, Milwaukee, WI; Ascension Columbia St. Mary's Milwaukee, Milwaukee, WI; Ascension Columbia St. Mary's Milwaukee, Milwaukee, WI; Ascension Columbia St. Mary's Milwaukee, Milwaukee, WI

## Abstract

**Patient Presentation (age range, injury details, relevant history):**

We used the case of a 49 year old male status post motor vehicle crash admitted with 47% TBSA with burns to face, head, neck, arms, torso, and legs to enact a unique quality improvement project. This patient had serial debridement and grafting surgeries over a LOS of 96 days.

**Clinical Challenges:**

Previous patients received rehabilitation at unaffiliated settings after discharge, and after our team noticed a pattern of suboptimal progress including a reduction in ROM, strength, functional mobility and independence, we identified poor communication standards between the acute setting and secondary treatment facilities. Our aim was to improve continuity of care between facilities to optimize burn survivor recovery outcomes.

**Management Approach:**

To guide our plan we identified areas hindering inter-facility communication: therapy instructions were buried in discharge summaries, latest data was not accessible, and no centralized burn rehabilitation resources were available to outside facilities. We then created an original rehabilitation discharge document (Fig. 1), taking into account the deficits we found upon previous patients' return and clearly describing the patient's current level of function, areas of deficit, expected progress, treatment plans/interventions, burn resources, and contact information. This tool was formally sent with our case study as he was discharged to an LTAC facility.

**Outcomes:**

This patient returned to our facility just two weeks after initial discharge with worsened deficits in functional mobility, cognition, range of motion, and strength despite the use of our first discharge document. The facility stated that they had not used the tool, either due to their therapists not knowing about its existence or being unable to find it. There were no noted attempts at communication from the receiving facility, making conclusions about our original documentation difficult to assess. We also could not share a formal letter of feedback as the original tool contained a lack of objective measurements to compare to the current status of the patient.

**Lessons Learned:**

Our initial implementation of the tool was flawed but invaluable in confirming the importance of quality communication between facilities and identifying areas of the discharge process that can be improved. Feedback from the facilities validated the importance of our quality improvement project. In response, we updated our document for enhanced communication, follow-up, and increased objective data for comparisons on patient progress, partially shown in Fig. 2.

**Applicability to Practice:**

The long term outcome of our patients is dependent on the level of care throughout the entire recovery process and when a patient is treated at a facility without burn trained therapists, high level and organized communication becomes paramount. Our tool is a cost-efficient, customizable, simple method to accomplish this.

